# Genomic Analysis of the Serratia marcescens Bacteriophage BUCT660

**DOI:** 10.1128/mra.00406-22

**Published:** 2022-07-14

**Authors:** Yahao Li, Pengjun Han, Mingfang Pu, Fei Li, Mengzhe Li, Xiaoping An, Lihua Song, Huahao Fan, Yigang Tong, Zeliang Chen

**Affiliations:** a Beijing Advanced Innovation Center for Soft Matter Science and Engineering, Beijing University of Chemical Technology, Beijing, China; b College of Life Science and Technology, Beijing University of Chemical Technology, Beijing, China; Portland State University

## Abstract

Here, we report the complete genome sequence of bacteriophage BUCT660, which comprises a linear double-stranded DNA (dsDNA) genome of 272,720 bp and a G+C content of 47%. BUCT660 contains 316 open reading frames and 2 tRNA-encoding genes. The results of transmission electron microscopy (TEM) indicate that BUCT660 is a member of the family *Caudooviricetes*.

## ANNOUNCEMENT

Serratia marcescens, a bacterium thought to be harmless to humans, survives in water and soil. But in recent years, more and more evidence has shown that Serratia marcescens can cause human infections and many diseases, such as bacteremia, endotoxin shock, and endocarditis (1, 2). Bacteriophages are widely found in nature, and lytic phages have the potential to treat bacterial diseases.

In this research, the pure Serratia marcescens 1373 clone (16S sequence shown in Table 1) from the China-Japan Friendship Hospital in Beijing, China, was isolated by streaking a sample onto an LB agar plate, and bacteriophage BUCT660 was isolated from the sewage of the same hospital. Phage BUCT660 was purified by five successive single-plaque isolations. The isolation and purification methods used for bacteriophage BUCT660 are based on the classic double-layer agar method (3). BUCT660 was further purified by CsCl density gradient centrifugation for transmission electron microscopy (TEM) and DNA preparation. The phage was visualized by TEM (JEM-1200EX; JapanTEM) at 80 kV (4). Phage genomic DNA was extracted using the proteinase K/SDS method (5). A 2 × 300-nucleotide (nt) paired-end DNA library was prepared using a NEBNext Ultra II DNA library prep kit for Illumina according to the manufacturer’s instructions (6). The Illumina MiSeq sequencing platform was used to sequence the whole genome of BUCT660 (7). In total, 2,098,054 raw reads were generated. The raw sequencing data quality was analyzed using the quality control software FastQC v0.11.5, and the data were filtered for low-quality reads and adapter regions using Trimmomatic v0.36 with default parameters (8). The generated high-quality reads were assembled using SPAdes v3.13.0 (9). The ends of the BUCT660 genome were determined using PhageTerm v3 (10). RAST was used to perform genome sequence annotations. All predicted open reading frames (ORFs) were manually checked using BLASTp against the NCBI nonredundant (nr) database; an E value of ≤0.05 was the cutoff used for identification (https://www.ncbi.nlm.nih.gov/) (11). tRNAscan-SE was used for the prediction of genes encoding tRNAs (http://lowelab.ucsc.edu/tRNAscan-SE/index.html) (12).

**TABLE 1:** 16S sequence for the Serratia marcescens 1373 clone

16S sequence
ACACATGCAGTCGAGCGGTAGCACAGGAGAGCTTGCTCTCTGGGTGACGAGCGGCGGACGGGTGAGTAATGTCTGGGAAACTGCCTGATGGAGGGGGATAACTACTGGAAACGGTAGCTAATACCGCATAACGTCGCAAGACCAAAGAGGGGGACCTTCGGGCCTCTTGCCATCAGATGTGCCCAGATGGGATTAGCTAGTAGGTGGGGTAATGGCTCACCTAGGCGACGATCCCTAGCTGGTCTGAGAGGATGACCAGCCACACTGGAACTGAGACACGGTCCAGACTCCTACGGGAGGCAGCAGTGGGGAATATTGCACAATGGGCGCAAGCCTGATGCAGCCATGCCGCGTGTGTGAAGAAGGCCTTCGGGTTGTAAAGCACTTTCAGCGAGGAGGAAGGTGGTGAACTTAATACGCTCATCAATTGACGTTACTCGCAGAAGAAGCACCGGCTAACTCCGTGCCAGCAGCCGCGGTAATACGGAGGGTGCAAGCGTTAATCGGAATTACTGGGCGTAAAGCGCACGCAGGCGGTTTGTTAAGTCAGATGTGAAATCCCCGGGCTCAACCTGGGAACTGCATTTGAAACTGGCAAGCTAGAGTCTCGTAGAGGGGGGTAGAATTCCAGGTGTAGCGGTGAAATGCGTAGAGATCTGGAGGAATACCGGTGGCGAAGGCGGCCCCCTGGACGAAGACTGACGCTCAGGTGCGAAAGCGTGGGGAGCAAACAGGATTAGATACCCTGGTAGTCCACGCTGTAAACGATGTCGATTTGGAGGTTGTGCCCTTGAGGCGTGGCTTCCGGAGCTAACGCGTTAAATCGACCGCCTGGGGAGTACGGCCGCAAGGTTAAAACTCAAATGAATTGACGGGGGCCCGCACAAGCGGTGGAGCATGTGGTTTAATTCGATGCAACGCGAAGAACCTTACCTACTCTTGACATCCAGAGAACTTANCAGAGATGNATTGGTGCCTTCGGGAACTCTGAGACAGGTGCTGCATGGCTGTCGTCAGCTCGTGTTGTGAAATGTTGGGTTAAGTCCCGCAACGAGCGCAACCCTTATCCTTTGTTGCCAGCGGTTCGGCCGGGAACTCAAAGGAGACTGCCAGTGATAAACTGGAGGAAGGTGGGGATGACGTCAAGTCATCATGGCCCTTACGAGTAGGGCTACACACGTGCTACAATGGCGTATACAAAGAGAAGCGACCTCGCGAGAGCAAGCGGACCTCATAAAGTACGTCGTAGTCCGGATTGGAGTCTGCAACTCGACTCCATGAAGTCGGAATCGCTAGTAATCGTAGATCAGAATGCTACGGTGAATACGTTCCCGGGCCTTGTACACACCGCCCGTCACACCATGGGAGTGGGTTGCAAAAGAAGTAGGTAGCTTAACCTTCGGGAGGGCGCTACCAC

**FIG 1:**
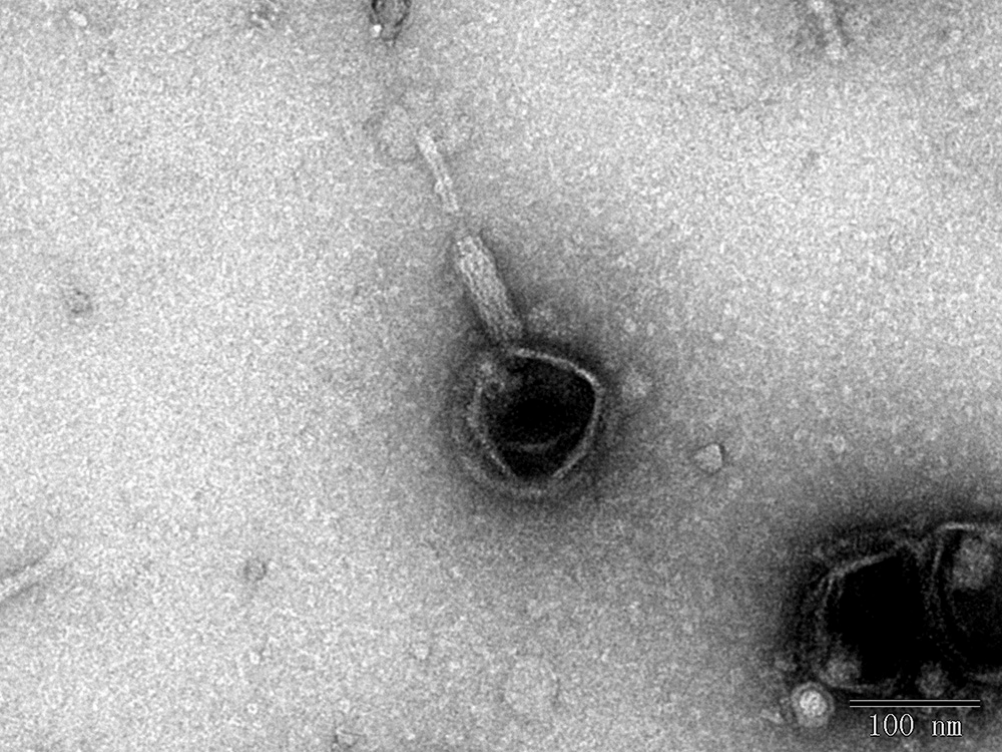
Transmission electron micrograph of Serratia marcescens phage BUCT660. Scale bar, 100 nm. The phage particles were negatively stained with 2% (wt/vol) phosphotungstic acid (pH 7.0) for 2 min and examined using a JEM-1200EX transmission electron microscope at an acceleration voltage of 80 kV. About 20 particles were measured to determine the size of the phage particles.

The TEM results showed that BUCT660 has an isometric head (121.56 ± 1 nm) and a contractile tail (166.88 ± 2 nm) (Fig. 1). According to the current International Committee on Taxonomy of Viruses classification system, it belongs to the family *Caudoviricetes*. BUCT660 has a linear double-stranded DNA (dsDNA) genome of 272,720 bp with a G+C content of 47% and terminally redundant ends. A total of 316 open reading frames were found in the genome of BUCT660, including 2 tRNAs, 236 genes annotated as hypothetical proteins encoding genes, and 78 with known functions. These known functional ORFs can be divided into five categories: lysis proteins, regulatory proteins, structural proteins, DNA packaging-related proteins, and replication-related proteins. A comparison of the genome sequence of BUCT660 with other sequences in the GenBank nr databases using BLASTn (the cutoff E value was ≤0.05) showed that there was no homologous phage of BUCT660 (13).

In summary, BUCT660 is a new member of the Serratia marcescens phages, with a large DNA genome. Interestingly, most of the open reading frames in its genome are not functionally annotated; further investigation of BUCT660 will be beneficial for better understanding the diversity of Serratia marcescens phages.

### Data availability.

The complete genome sequence of phage BUCT660 with annotations was submitted to GenBank under the accession number OK040170. The raw sequence of phage BUCT660 was submitted to the SRA under the accession number SRX14325425 and the BioProject accession number PRJNA810917.
